# Breast Cancer: Surgery at the South Egypt Cancer Institute

**DOI:** 10.3390/cancers2031771

**Published:** 2010-09-30

**Authors:** Ahmed A.S. Salem, Mohamed Abou Elmagd Salem, Hamza Abbass

**Affiliations:** 1 Surgical Oncology and Endoscopy unit, South Egypt Cancer Institute, Assiut University, Egypt; 2 Lecturer of Surgical Oncology, South Egypt Cancer Institute, Assiut University, Egypt; E-Mail: salem641972@yahoo.com; 3 Lecturer of Radiotherapy, South Egypt Cancer Institute, Assiut University, Egypt; E-Mail: hamza_assiut@yahoo.com

**Keywords:** breast cancer, figures, Egypt

## Abstract

Breast cancer is the most frequent malignant tumor in women worldwide. In Egypt, it is the most common cancer among women, representing 18.9% of total cancer cases (35.1% in women and 2.2% in men) among the Egypt National Cancer Institute’s (NCI) series of 10,556 patients during the year 2001, with an age-adjusted rate of 49.6 per 100,000 people. In this study, the data of all breast cancer patients presented to the surgical department of the South Egypt cancer Institute (SECI) hospital during the period from Janurary 2001 to December 2008 were reviewed .We report the progress of the availability of breast cancer management and evaluation of the quality of care delivered to breast cancer patients. The total number of patients with a breast lump presented to the SECI during the study period was 1,463 patients (32 males and 1431 females); 616 patients from the total number were admitted at the surgical department .There was a decline in advanced cases. Since 2001, facilities for all lines of comprehensive management have been made accessible for all patients. We found that better management could lead to earlier presentation, and better overall outcome in breast cancer patients.The incidence is steadily increasing with a tendency for breast cancer to occur in younger age groups and with advanced stages.

## 1. Introduction

Breast cancer is the most frequent malignant tumor in women worldwide. The incidence and mortality rates among females vary among countries but are steadily increasing worldwide [1,2,3].

Ancient Egyptians were the first to note breast cancer more than 3,500 years ago. Both the Edwin Smith and George Ebers papyri contain descriptions of conditions that are consistent with modern descriptions of breast cancer [4].

In Egypt, breast cancer is the most common cancer among women, representing 18.9% of total cancer cases (35.1% in women and 2.2% in men) among the Egypt National Cancer Institute (NCI) series of 10,556 patients during the year 2001 [5], with an age-adjusted rate of 49.6 per 100,000 population. However, this represents hospital-based data of referral tertiary centers and does not represent all breast cancer cases in Egypt. The population-based cancer registry of Egypt in Ghrabiah [6]. The median age at diagnosis is one decade younger than in countries of Europe and North America and most patients are premenopausal [7].

Important advances have been made in the strategies of early detection and of therapeutic interventions of breast cancer which may contribute to a more favorable development of its natural history.

Inappropriate therapy is a major aspect of inequity in health that exists in many developing countries. Low health budgets may not be the only causative factor. Other factors such as the limited diffusion of practice guidelines and lack of continuous education of health-care providers may also be implicated in such inappropriate care. However, the quality of breast-cancer management seems to be unsatisfactory in both developed and developing countries [6].

The proportion of women receiving appropriate primary therapy in the USA fell from 88% for the period of 1983–1989 to 78% by the end of 1995. This decline was observed in all subgroups of age, race, stage and population density. The proportion undergoing an inappropriate form of mastectomy was 2.7%. Furthermore, the proportion undergoing an inappropriate form of breast conserving surgery increased from 10% in 1989 to 19% by the end of 1995 [7].

Another study performed in Italy during the period 1988–1989 reported that 38% of patients had an inappropriate form of surgery with more than two-thirds being accounted for by the use of unnecessary mutilating Halsted mastectomy. Substantial geographical variations emerge in the overall rates of inappropriate treatment (range 12–48%) which are not substantially affected by allowance for imbalances inpatient and hospital-related variables.

The current work was undertaken to study and evaluate the quality of therapeutic care delivered to breast-cancer patients in a representative sample from the university hospital in Assiut, Egypt.

## 2. Materials and Methods

Information for the present work, concerning health care delivered to breast-cancer patients, was collected over the period January 2001 to December 2008 in the South Egypt Cancer Institute, Assiut University.

Assiut University Hospital and Hospital of South Egypt Cancer Institute are the only hospitals providing radiotherapy services to the majority of cancer patients, where the expenses are totally covered by the government. The radiotherapy services in these hospitals include Cobalt machines, linear accelerators and simulators.

Clinical staging (designated cTNM or TNM) according to the fifth edition of the AJCC manual for cases between 2001–2002, while the sixth edition is used for cases from 2003 and after, is based on all information available prior to when the first definitive treatment was used and includes the findings on physical examination, imaging studies, operative findings, and pathologic examination of the breast or other tissues [8].

Imaging studies were performed for all patients prior to surgery for staging purposes and then every six months for follow-up. The imaging studies included: chest X-ray, abdominal ultrasongraphy and bone scan. Operative findings that are appropriate for clinical staging include the size of the primary tumor, the presence of chest wall invasion and the presence or absence of regional or distant metastases.

Operative findings are guided by the results of imaging studies. Concerning the menopausal status, women with absence of the menstrual cycles for six successive months were considered post-menopausal.

## 3. Results

The study population consisted of a total of 616 patients (32 males and 584 females) with a mean age of 46.5 years, ranging from 19 years old to 89 years old. 40 patients were admitted during year 2001 with a steady increase in incidence up to 124 patients during year 2008. Almost half of the cases fell in the age category 40–59 years where the most frequent age group was 40–49 years. 81.9% were married. From the total number of patients presented to our surgical department, 23 cases proved to be benign and most of them were in the age group of 20–29 years.

In regard to stage distribution, 70 (11%) patients had stage I cancer progression, 240 (39%) patients had stage II, stage III 152(25%), and stage IV 150 (25%). Fifty percent (50%) of our patients had advanced stages (III and IV) at presentation. Informed consent was taken before surgery; no patient refused surgery for breast cancer. In regard to surgical management, Patey's modified radical mastectomy was the most commonly performed regardless of clinical stage (419 cases from the total number). The frequency and the type of all surgical procedures are listed in Table 1.

**Table 1:** Frequency and type of surgical procedures.

Operation	Number of patients	%
Modified radical mastectomy(MRM)	419	68%
Radical mastectomy	9	1.5%
MRM with reconstruction with TRAM	5	0.8%
MRM with reconstruction with LDF	6	0.97%
Breast conservative surgery (lumpectomy and axillary evacuation)	38	6.2%
Skin sparing mastectomy	2	0.32%
Simple mastectomy	5	0.8%
Excisional biopsy	85	13.8%
Incisional biopsy	35	5.7%
Local excision of recurrent nodules	7	1.13%

From 2001 to 2008, increased rate of breast conservation was noted and there was a decline in the use of the Hatested mastectomy.

**Figure 1:**
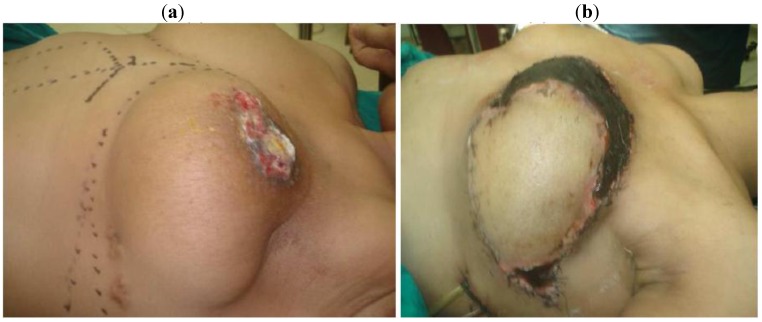
(**a**) Female patient 27 years with ulcerating local recurrence after breast conservative surgery; (**b**) surgery reconstruction with TRAM flap after excision.

**Figure 2:**
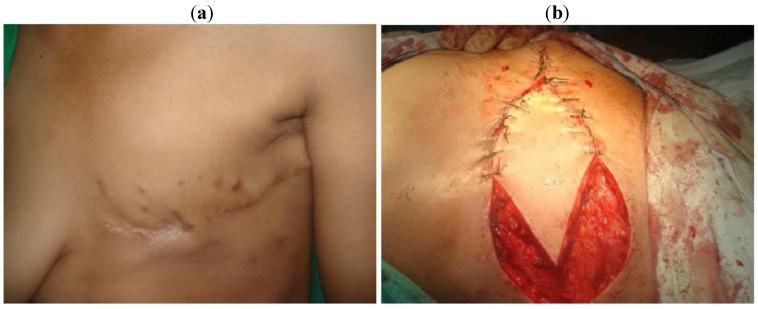
(**a**) female patient 45 years with recurrent skin nodules after MRM; (**b**) Reconstructed with LDF after excision.

Immediate breast reconstruction with TRAM (Figure 1a,b) or LDF (Figure 2a,b) was used in 11 cases; however in two of these cases the flap was used to cover the defect after local excision of local recurrence However, there was a significant association between the clinical stage and the type of surgical operation performed, as well as postoperative radiotherapy administration. Halsted radical mastectomy was performed for stage IV patients and male patients with breast cancer.

Systemic therapy was given mainly as an adjuvant therapy in 95.1% of cases with 2.9% receiving it as palliative treatment in stage IV, while only 2% of cases received chemotherapy as neoadjuvant treatment.

Radiotherapy was not administered prior to surgery. It was administered postoperatively as an adjuvant in 96.5% of cases and for palliation to 3.5% of cases.

## 4. Discussion

In the present study, the average age at diagnosis was 46.5 years, which is one decade younger than that in most Western countries. Giles *et al*. reported an average age of 56 years for breast cancer patients in Victoria, Australia [9].

As reported by Mohamed Metwaly *et al*., the median age incidence of Kuwaitis breast cancer patients was 45 years, which is close to that of Egyptian patients [10].

It is of interest, however, that the rates in the very young age groups in Egyptians, but not in Jordanians or Israeli Arabs, were similar to the rates in Israeli Jews, which are among the highest in the world. Given the fact that the Egyptian registry covers only part of the Egyptian population, a remote possibility that some of these rates reflect a selection into the study cohort of younger women cannot be ignored [11]. Nevertheless, a study of immigrants from the Middle East to Australia did indicate that the Egyptian women had the highest breast cancer rates of all Middle Eastern immigrants [12].

Patey's mastectomy is usually the most common surgical procedure adopted in the surgical department of the South Egypt Cancer Institute, Egypt. The limited diffusion of conservative surgery for the management of early breast cancer cases is explained by the fact that clinicians are not confident of their patients’ compliance with planned radiotherapy regimens and regular follow-up. Other reasons for the preference of mastectomy include the fear and inconvenience of radiotherapy and a perception that survival might be diminished if mastectomy is not done.

However, the recent practice guidelines state that women with early stage invasive breast cancer who are candidates for breast conservation therapy should be offered the choice of either breast conservation therapy or modified radical mastectomy. The choice is, therefore, an individual one for the patient, and all patients should be fully informed of the options, including the risks and benefits of each procedure. Women need to be informed that breast irradiation is part of the procedure of breast conservation therapy. In addition, women need to be aware of the potential need for further surgery if excision margins are positive [13].

However, there is evidence of a variation in the practice of radiotherapy in the management of women with early stage breast cancer even in developed countries, which appear to arise from scientific uncertainty [14].

Therefore, while there has been a decreasing tendency to perform mastectomy, falling from 52% to 28% in favor of conservative breast surgery during the 1980s, the proportion of mastectomy among our cases ranged from 85.2% to 100% according to clinical stage and health facility.

However, the higher performance of mastectomy in teaching hospitals is consistent with other results reported in the literature [15]. This study could not identify the proportion of patients in whom conservative surgery could have been performed.

For patients who are candidates for a mastectomy, skin sparing mastectomy or nipple sparing mastectomy with immediate autologous reconstruction are oncologically safe techniques.

Adjuvant radiotherapy decreases the esthetic results even after a longer period of time [16]. The majority of cases received postoperative radiotherapy. Postoperative radiotherapy was given to all patients, as practice guidelines recommend that irradiation should be an integral part of multimodality management of breast cancer due to the significant improvement in loco-regional control and overall survival. Our data show that despite less than 5% of patients having conservative surgery, 92% of patients had postoperative radiotherapy following mastectomy, while only a proportion of patients having mastectomy required such additional radiotherapy.

There are concerns about poor patient compliance and limited facilities for radiotherapy following breast-conservative surgery in our community. Moreover, the wide-spread use of post-mastectomy radiotherapy to a large proportion if patients will reduce their risk of local recurrence even if the patients do not attend follow-up.

The fact that less than 11% of our cases were stage I at first diagnosis indicates deficits in health education in women in our society about the risk of developing breast cancer. Women ought to be fully informed about the importance of breast self-examination (BSE) and attending routine annual mammography from 35 years of age or even younger among women having first degree relatives with breast cancer. It is well recognized that a patient's chances of disease recurrence and death depend on the number of cancer-positive regional lymph nodes at the time of diagnosis [17].

A recent study reported that only 13.3% of breast cancer patients in Egypt had ever had mammography performed as a diagnostic procedure. This study also revealed that the patient-related diagnostic delay (an interval between first symptom and first medical consultation of 43 months) was found in 38.1% of cases [18]. Furthermore, only 10.4% of Egyptian women had ever performed BSE [19]. These previous reports have uncovered marked deficiencies in educating Egyptian women in regard to the need for mammography and BSE.

## 5. Conclusion

We can conclude that Patey's modified radical mastectomy, rather than conservative surgery, is the most commonly performed surgical operation for early stages of breast cancer. The incidence of breast cancer is steadily increasing with a tendency to occur in younger age groups and with advanced stages in Egypt. Since breast conservation protocols and breast reconstruction yield results similar to mastectomy, its use should be extended. Furthermore, money is often spent inappropriately on excessive unnecessary follow-up investigations such as scans and chest X-rays. Cost-benefit studies are, therefore, warranted in order to rationalize health expenditure aiming at a better health care for breast cancer patients in Egypt.

We can also add that there is defective screening and health education for women, and targeted intervention strategies are, therefore, warranted to improve early detection and hence better prognosis of breast cancer cases in our community.
